# Dendrimers as Soft Nanomaterials for Electrochemical Immunosensors

**DOI:** 10.3390/nano9121745

**Published:** 2019-12-07

**Authors:** Alfredo Sánchez, Anabel Villalonga, Gonzalo Martínez-García, Concepción Parrado, Reynaldo Villalonga

**Affiliations:** Nanosensors & Nanomachines Group, Department of Analytical Chemistry, Complutense University of Madrid, 28040 Madrid, Spain; anabelvillalonga.inf@gmail.com (A.V.); gmarti01@ucm.es (G.M.-G.); cparrado@ucm.es (C.P.)

**Keywords:** antibody, dendrimer, electrochemical immunosensor, polyamidoamine, poly (propylene imine)

## Abstract

Electrochemical immunosensors are antibody-based affinity biosensors with a high impact on clinical, environmental, food, and pharmaceutical analysis. In general, the analytical performance of these devices is critically determined by the materials and reagents used for their construction, signal production and amplification. Dendrimers are monodisperse and highly branched polymers with three-dimensional structures widely employed as “soft” nanomaterials in electrochemical immunosensor technology. This review provides an overview on the state-of-the-art in dendrimer-based electrochemical immunosensors, focusing on those using polyamidoamine and poly (propylene imine) dendrimers. Special emphasis is given to the most original methods recently reported for the construction of immunosensor architectures incorporating dendrimers, as well as to novel sensing approaches based on dendrimer-assisted signal enhancement strategies.

## 1. Introduction

Electrochemical biosensors and technologies associated with these sensing systems are emerging and fast-growing research areas in basic and applied chemistry [1,2,3,4]. They are also valuable commercial products with an estimated global market of $12.8 billion in 2018 and an expected growth over $23.7 billion for 2022, at a CAGR of 9.7% [5].

These sensors have wide applications in agriculture, environmental monitoring, food safety, industrial processes, forensic analysis, research laboratories, biotechnology, and homeland security & biodefense [6,7,8]. However, the major impact of electrochemical biosensor technology has been in biomedical areas, in which these analytical devices are used for the clinical and home diagnosis of relevant diseases [9,10]. Electrochemical biosensors also constitute promising tools for the development of point-of-care sensing systems able to permit real-time and remote health monitoring [11]. Moreover, these analytical devices have been envisioned to play a significant role in newer end uses, such as automotive and aerospace industry.

In general, electrodechemical biosensors are self-contained, integrated analytical devices, consisting of a biological recognition element in direct contact or incorporated with an electrochemical transduction element, which converts a biological recognition process into a useable output signal [12]. These biosensors can be classified into two major categories according to the biorecognition principle:

(1) Catalytic biosensors, which are those using a biorecognition element able to both recognize and catalyze the transformation of the target analyte.
(2) Affinity biosensors, in which the biorecognition element recognizes the target analyte through an affinity-based mechanism but does not lead to its chemical transformation.

This second category includes the electrochemical immunosensors in which the biomolecular receptor is an antibody. These affinity biosensors have received considerable research attention due to their unique properties, combining the high target affinity and specificity provided by the antibody-mediated biorecognition process with the high sensitivity, low detection limit, affordability, small size, and portability associated with electrochemical transducers [13,14].

In general, both the biorecognition processes and the electrochemical detection take place at the interface between the samples solution and the electrode surface on which the biological recognition element is usually located. For this reason, the design of a suitable electrode surface plays a crucial role in the performance of electrochemical biosensors. In this sense, electrode surface architectures able to provide a favorable environment for the stable, reproducible and high yield immobilization of the biorecognition elements, preserving their biological activity, and allowing the proper occurrence of the electrocatalytic and electron transfer processes are desired [15,16,17,18].

Several strategies have been employed to prepare electrodes with suitable surface characteristics, including coating with self-assembled monolayers of low and high molecular weight compounds, functionalization with natural and synthetic polymers, and modification with micro- and nanosized materials [1,2,3,19,20,21]. In this context, nanomaterials have demonstrated to be excellent building blocks for the design of original and affordable 3D assemblies on sensor surfaces, allowing improvement of the analytical and stability properties of electrochemical biosensors [1,2,3,4].

During the last decades, nanomaterials engineering has provided a large variety of “hard” nanomaterials for biosensors construction, including metal and metal oxide nanoparticles, carbon nanotubes, graphene, porous films, etc. [22]. In addition, relevant attention has been devoted to use dendrimers as “soft” nanomaterials to tailor the chemical and physical properties of electrode surfaces. These macromolecules have also demonstrated to be useful tools to produce and amplify the analytical signal in novel electrochemical biosensors [23,24,25].

**Figure 1:**
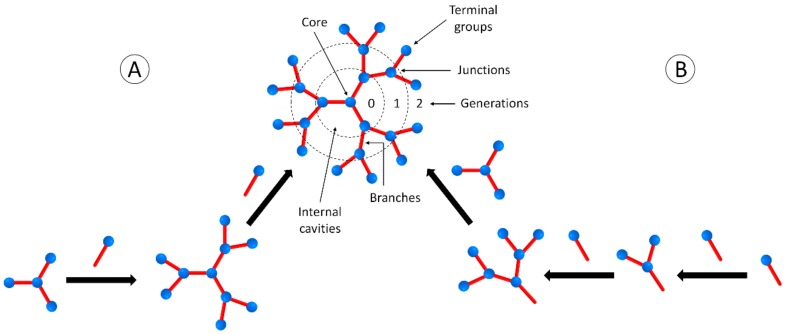
Structural parameters of a dendrimer molecule prepared through divergent (**A**) and convergent (**B**) methods of synthesis. Blue circles: functional groups, red bars: chemical branches.

Dendrimers are monodisperse and nanosized synthetic polymers with a regular and highly branched three-dimensional structure [26,27]. These macromolecules are usually prepared by convergent and divergent methods of synthesis, through an iterative sequence of reaction steps in which each additional iteration leads to a higher generation material with increased terminal groups at the molecule surface. Figure 1 illustrates both synthetic strategies, as well as the main structural parameters for a dendrimer molecule.

These hyperbranched soft nanomaterials have unique physicochemical properties such as structural uniformity, globular or ellipsoidal shape, nanometric size and monodispersity, high density of functional groups at the surface, and high permeability of the internal cavities [26]. The possibility to manipulate such properties by controlled chemical transformation or tailor-made design of dendrimers with specific composition also offers versatile possibilities to these polymers as functional 3D scaffolds for constructing nanostructured designs at the electrode surfaces [28,29,30]. Dendrimers have also been proved to be useful soft nanocontainers for the supramolecular encapsulation of a great variety of compounds and small nanoparticles in their interior through electrostatic or hydrophobic interactions to form host–guest complexes [31,32,33]. This property has been elegantly exploited in biosensor technology to favor electrocatalytic events as well as for signal production and amplification purposes.

A great variety of dendrimers have been synthesized since the pioneer works of Vögtle [34], Denkewalter [35], Tomalia [36], and Newkome [37]. This includes polyamidoamine (PAMAM), poly (propylene imine) (PPI), polyesters, poly (ester amides), polyethers, poly (ether amides), polyalkanes, polyphenylenes, poly (phenylacetylenes), polysilanes, and phosphorus dendrimers, as well as several coordinated dendrimers [38]. Some of these hyperbranched polymers, with different core structures, terminal groups, generation, and grade of purity, are produced at industrial scale and are commercially available, which makes their use in biosensor construction easy.

The aim of this review is to discuss the most relevant works on the application of dendrimers in electrochemical immunosensors during the last decade. In this sense, the coverage of this review will include outstanding examples concerning the use of these hyperbranched polymers for transduction and signal production and amplification in these affinity biosensors, with special attention to PAMAM and PII dendrimers. Detailed descriptions of the synthesis, physicochemical characteristics, binding properties, and application of dendrimers in other fields than bioanalytical electrochemistry are beyond the scope of this review and have been previously covered by other authors.

## 2. Dendrimers as Transduction Elements in Electrochemical Immunosensors

**Figure 2:**
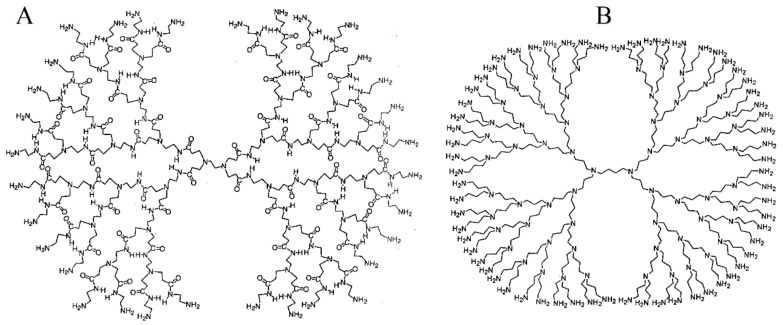
Structure of ethylenediamine core PAMAM G-4 (**A**) and butylenediamine core PPI G-5 (**B**) dendrimers [42].

There are several reasons to use dendrimers as modification agents for electrodes to serve as transduction elements in electrochemical immunosensors, such as:

- Dendrimers have a great density of chemical groups located at their periphery [26], which can promote their stable attachment to the electrode surface. These chemical functionalities can also be employed as linking points for the immobilization of antibodies through covalent or non-covalent interaction.
- These hyperbranched polymers are nanosized macromolecules with a permeable globular or ellipsoidal structure, which causes low barrier effects to the diffusion of electroactive substances at the electrode surface.
- The well-defined shape and composition, relative conformational rigidity, as well as the presence of high density chemical groups at the dendrimer surface allow their use as valuable building blocks for the controlled design of molecularly organized monolayers and multilayers architectures on different surfaces [39].
- Dendrimers can be rationally tuned to promote electrocatalytic and charge transfer processes at the electrode surface by proper encapsulation or covalent binding of small metal nanoparticles and electron transfer mediators [31,33].
- Several dendrimers, including the commercially available PAMAM and PPI (Figure 2), show high compatibility with proteins such as antibodies, due to their hydrophilic surfaces allowing preservation of the biologically active conformation of these bioreceptors upon immobilization/association [40,41].
- Finally, but not less important, there is the possibility to combine dendrimers with a great variety of nanomaterials and polymers to design novel hybrid materials for electroanalytical applications.

**Figure 3:**
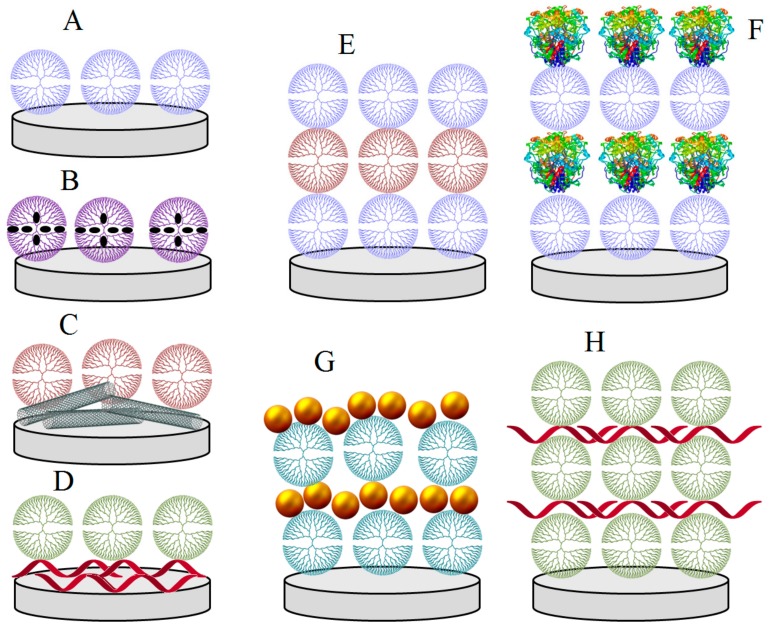
Assemblies of dendrimers on electrode surfaces: (**A**) molecularly organized dendrimer monolayer; (**B**) monolayer of metal nanoparticle-decorated dendrimers; (**C**) dendrimer layered on nanomaterial-modified surface; (**D**) dendrimer layered on polymer-coated surface, and layer-by-layer assemblies of (**E**) dendrimer/dendrimer, (**F**) dendrimer/protein, (**G**) dendrimer/nanoparticles, and (**H**) dendrimer/polymer bilayers.

Different assembly strategies can be adopted to construct dendrimer-based 3D layer arrangements on an electrode surface, including molecularly organized monolayers and a great variety of hybrid layers when combined with polymers and nanomaterials. These fractal-like macromolecules can also be employed as building blocks for the preparation of ordered layer-by-layer architectures with other dendrimers, proteins, polymers and “hard” nanomaterials. Some of these possible arrangements are illustrated in Figure 3.

The last decade has been characterized by the publication of a great numbers of reports dealing with the use of dendrimers to modify electrodes for the construction of electrochemical immunosensors. Here we do not intend to make an inventory of all reports concerning this area of biosensor technology, but to provide a general sight state-of-the-art on the use of these hyperbranched polymers as functional soft nanomaterials for electrode design.

PAMAM dendrimers are, by far, the hyperbranched polymers most exhaustively employed in electrochemical biosensors, and many authors have taken advantage of the use of electrodes modified with monolayers of this polymer to assemble original immunosensor architectures. For example, Akter and co-worker described a simple immunosensor design by covalently layering PAMAM G-3.0 dendrimer with surface terminated succinamic acid groups on Au electrodes coated with a self-assembled monolayer of 6-mercaptohexanoic acid modified with 3, 3′, 5, 5′-tetramethyl benzidine [43]. Further immobilization of an anti-cardiac troponin I monoclonal antibody allowed the impedimetric detection of up to 11.7 fM of this protein.

In other work, Giannetto and co-workers described the electrodeposition of gold nanoparticles (AuNP) on glassy carbon electrodes (GCE) and further functionalization with 2-aminoethanethiol and a covalently attached self-assembled monolayer of PAMAM G-1.5 dendrimer [44].

**Figure 4:**
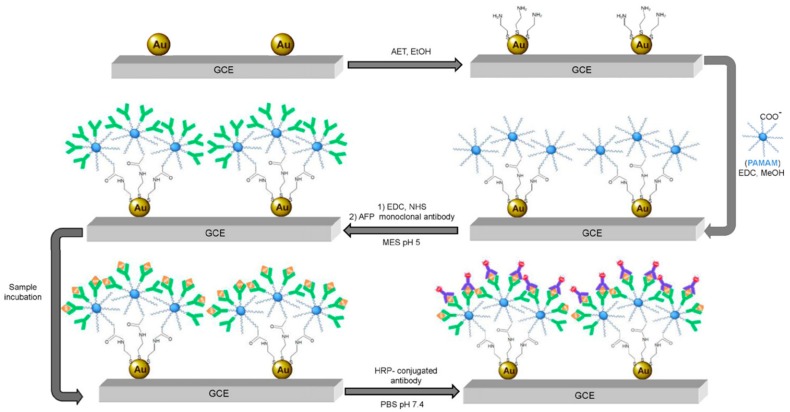
Schematic representation of the protocol developed for the fabrication of the immunosensor for α-fetoprotein. In green: anti-α-fetoprotein monoclonal antibody, in yellow: α-fetoprotein, in blue and red: HRP-conjugated antibody. Adapted with permission from Ref. [44], copyright Elsevier B.V., 2011.

As is illustrated in Figure 4, this surface was employed as support for the covalent immobilization of an anti-α-fetoprotein monoclonal antibody to design a sandwich-type immunosensing assay for this cancer biomarker. The resulting immunosensor was able to detect up to 3 ng/mL α-fetoprotein and was successfully validated with human serum samples. This research group further employed similar electrode architecture with layered PAMAM molecules to covalently attach 2,4,6-trinitrotoluene-ovalbumin [45] and atrazine-bovine serum albumin [46] conjugates to develop two different competitive amperometric immunosensors for the target explosive and herbicide, respectively.

**Figure 5:**
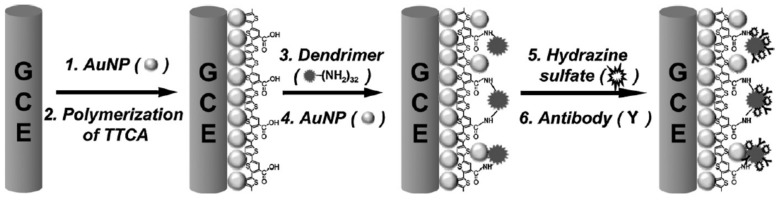
Schematic representation of the protocol developed for the fabrication of the immunosensors for Annexin II or MUC5AC. Adapted with permission from Ref. [47], copyright Elsevier B.V., 2009.

With the aims to construct reliable biosensor devices for early diagnosis of lung cancer, Kim and co-workers covalently layered PAMAM G-3.0 dendrimers on GCE coated with electrodeposited AuNP and electropolymerized poly-terthiophene carboxylic acid [47]. As illustrated in Figure 5, this electrode surface was then decorated with colloidal AuNP and further functionalized with hydrazine and a specific antibody for Annexin II or MUC5AC. These electrodes were employed to detect the lung cancer biomarkers through a competitive immunosensing assay, implying the use of glucose oxidase-labeled analytes. This sensing approach takes advantage of the use of glucose as an enzyme substrate and hydrazine as a catalyst for the reduction of H_2_O_2_ generated by the glucose oxidase-mediated reaction.

**Figure 6:**
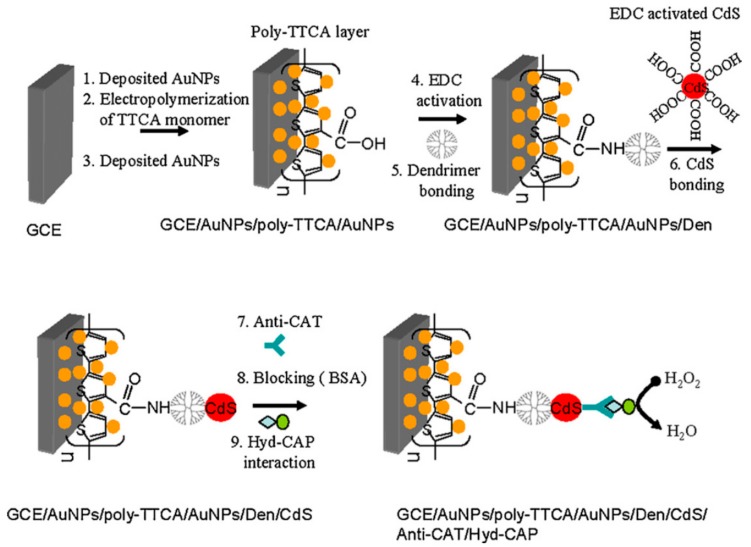
Schematic representation of the protocol developed for the fabrication of the competitive immunosensors for chloramphenicol. In yellow: Au nanoparticles, in red: CdS nanoparticles, in greenish blue: anti-chloramphenicol antibody, in light blue-green: chloramphenicol-hydrazine conjugate. Adapted with permission from Ref. [48], copyright Elsevier B.V., 2010.

In a further research, this group employed a similar electrode modified with AuNP, the conducting polymer poly-terthiophene carboxylic acid and PAMAM G-4.0 to attach CdS nanoparticles as sensitivity enhancers [48]. Further immobilization of a specific antibody for chloramphenicol allowed detection of this antibiotic though a competitive assay using a chloramphenicol–hydrazine adduct as a competitive target and signal generator through the hydrazine-catalyzed reduction of H_2_O_2_ at the electrode surface, as is shown in Figure 6. This immunosensor was able to detect chloramphenicol from 50 pg/mL to 950 pg/mL, with a detection limit of 45 pg/mL.

Namgung and co-workers reported other examples of the use of dendrimer layers with conductive organic materials for immunosensor construction [49]. In this sense, gold electrodes were first coated with a conductive self-assembled monolayer of the oligophenylethynylenethiol 4-(2-(4-(acetylthio)phenyl)ethynyl) benzoic acid, and a monolayer of PAMAM dendrimer was further attached via a carbodiimide-coupling reaction. Covalent immobilization of an anti-PSA antibody allowed the development of a sandwich-type immunosensing assay. Regrettably, relevant analytical figures of merit of this immunosensor were not provided in the published paper.

Another example of a dendrimer-layered immunosensor architecture was described by Lin and co-workers, which covalently attached PAMAM G-2.0 dendrimer on glassy carbon electrodes previously coated with an electrodeposited film of graphene/chitosan to construct an amperometric immunosensor for benzo[a]pyrene [50]. In addition, these authors constructed a competitive immunosensor for the same analyte by using Au electrodes coated with electropolymerized 2-amino-5,2′:5′2′′-terthiophene and PAMAM G-2.0 dendrimer as transducers, and methylene blue@SiO_2_ core@shell nanoparticle loaded with horseradish peroxidase and a secondary antibody as the labeling element [51].

A more complex sensor design was reported by Wang and co-workers, by constructing a AuNP/PAMAM G-4.0/AuNP layer-by-layer assembly on polyvinyl chloride membranes [52]. The modified membrane was used to immobilize a polyclonal antibody for the prostate-specific antigen (PSA), and the resulting immunosensing interface was deposited on the surface of Ag/AgCl electrode for the label-free potentiometric determination of the cancer biomarker. This simple and portable device was able to detect up to 0.1 ng/mL PSA and was successfully validated in human serum samples.

Tang and co-workers reported an original label-free immunosensing approach by using PAMAM dendrimer-layered electrodes as the transducer. The assembly of this immunosensor implied the sequential modification of Au electrodes with PAMAM G-4.0 dendrimers, core@shell SiO_2_@Au nanoparticles and a ferrocene-labeled anti-IgG monoclonal antibody. Interestingly, the authors used the enzyme glucose oxidase as a blocking reagent instead of other commonly employed proteins such as bovine serum albumin or casein. The sensing mechanism relied on the formation of the antibody-antigen complex, causing a barrier to the diffusion of glucose to the immobilized enzyme, and accordingly, a decrease in the voltammetric signal associated to the enzyme-mediated catalytic oxidation of this monosaccharide [53].

PAMAM dendrimers have been largely employed to produce hybrid nanomaterials for electrochemical immunosensing. A common approach is based on the encapsulation of catalytic metal nanoparticles into the dendrimer inner space and the use of the resulting nanohybrid as a transducer element. As an example, Tang and co-workers prepared a PAMAM G-4.0 dendrimer decorated with Au nanoparticles (AuNP) to construct a competitive immunoassay for the highly sensitive electrochemical determination of brevetoxin B in food samples [54].

**Figure 7:**
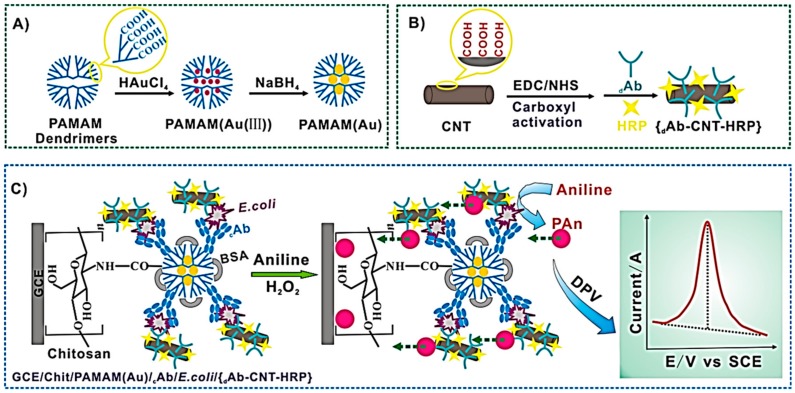
Schematic representation of the preparation of (**A**) PAMAM-Au nanoparticles hybrid material (PAMAM (Au)) and (**B**) carbon nanotubes functionalized with peroxidase and antibody ({dAb-CNT-HRP}). (**C**) Biosensing approach for E. coli detection using a sandwich immunoassay and electrochemical measurement of enzymatically produced polyaniline. Adapted with permission from Ref. [55], copyright Elsevier B.V., 2016.

A similar nanohybrid of AuNP encapsulated into PAMAM G-2.5 dendrimers was prepared and covalently attached to the surface of chitosan-coated glassy carbon electrodes to further immobilize an anti-*E. coli* monoclonal antibody [55]. As is illustrated in Figure 7, this electrode was employed to construct a sandwich-type assay for *E. coli* detection by using multi-walled carbon nanotubes functionalized with horseradish peroxidase and anti-*E. coli* antibody as labeling and signal amplification elements. The authors here proposed an original sensing approach, based on the addition of aniline and the electrochemical measurement of polyaniline produced through a peroxidase-catalyzed reaction.

More recently, Shen and co-workers coated Au electrodes with chitosan, a nanohybrid of AuNP/PAMAM G-4.0 dendrimer, a ferrocene-modified ionic liquid and a layer of AuNP [56]. Further immobilization of a monoclonal antibody for α-fetoprotein allowed the label-free detection of this cancer biomarker. The biosensing approach here proposed relied on the reduction of the voltammperometric response of the ferrocenyl groups attached to the ionic liquid upon affinity recognition of the biomarker. The rationale of using the dendrimer nanohybrid as a “bridge” reagent in this immunosensor architecture was justified by its capacity to retain more ionic liquids containing ferrocenyl groups on the electrode surface.

**Figure 8:**
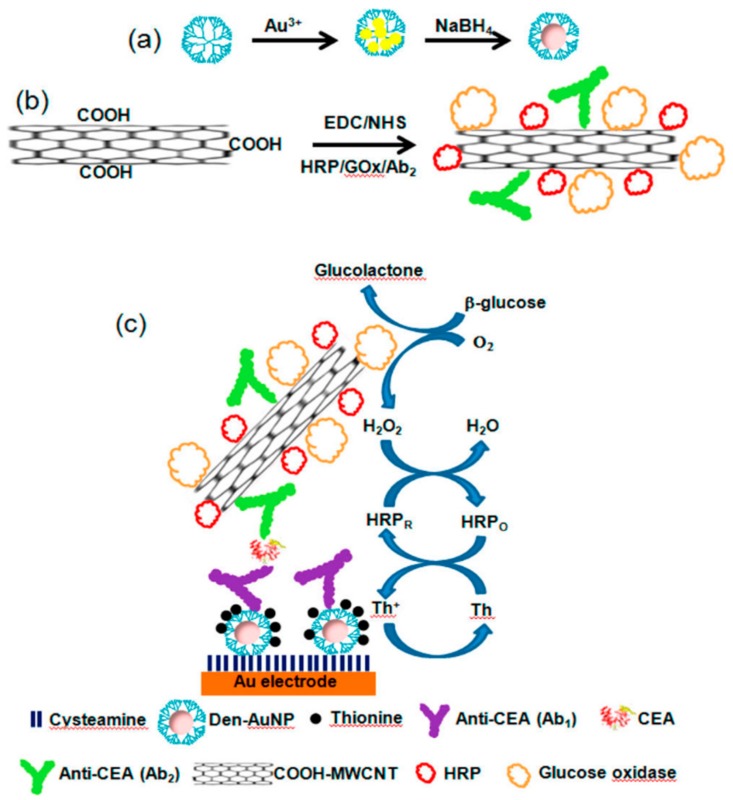
Schematic representation of the preparation of (**a**) PAMAM-Au nanoparticles hybrid material and (**b**) polyfunctionalized carbon nanotubes. (**c**) Biosensing approach for CEA detection using a sandwich immunoassay. Adapted with permission from Ref. [57], copyright American Chemical Society, 2013.

To construct a sensitive immunosensor for the carcinoembryonic antigen (CEA), Jeong and co-workers employed a nanohybrid of AuNP-encapsulated PAMAM G-3.0 dendrimer with surface-terminated succinamic acid groups to immobilize the specific antibody and thionine as electrochemical mediators [57]. This functionalized nanohybrid was used as a transduction element on Au electrodes to assemble a sandwich-type electrochemical immunoassay for this cancer biomarker by using multi-walled carbon nanotubes polyfunctionalized with an anti-CEA secondary antibody and the enzymes glucose oxidase and horseradish peroxidase as electrochemical labels. This original assembly, that is represented in Figure 8, allowed the highly selective detection of CEA in the range of 10.0 pg/mL to 50.0 ng/mL with a detection limit of 4.4 pg/mL.

As an example of another metal nanoparticles-encapsulated dendrimer for immunosensor construction, Singal and co-workers prepared a PAMAM G-4.5 dendrimer loaded with Pt nanoparticles, and this nanohybrid was electrodeposited on the surface of carbon screen-printed electrodes [58]. Covalent immobilization of a specific anti-human cardiac troponin-I monoclonal antibody allowed the impedimetric detection of this biomarker in the range of 1.0 pg/mL to 100 ng/mL.

Carbon nanomaterials have also been employed to prepare PAMAM-based nanohybrids for electrochemical immunosensors. As an example, Gao and co-workers reported a novel and one-step microwave-assisted pyrolytic protocol to prepare PAMAM G-2.0 dendrimer capped-carbon dots (PAMAM-CDs), which were further employed as reducing and capping agents for the formation of PAMAM-CDs/Au nanocrystal hybrid nanomaterial [59].

**Figure 9:**
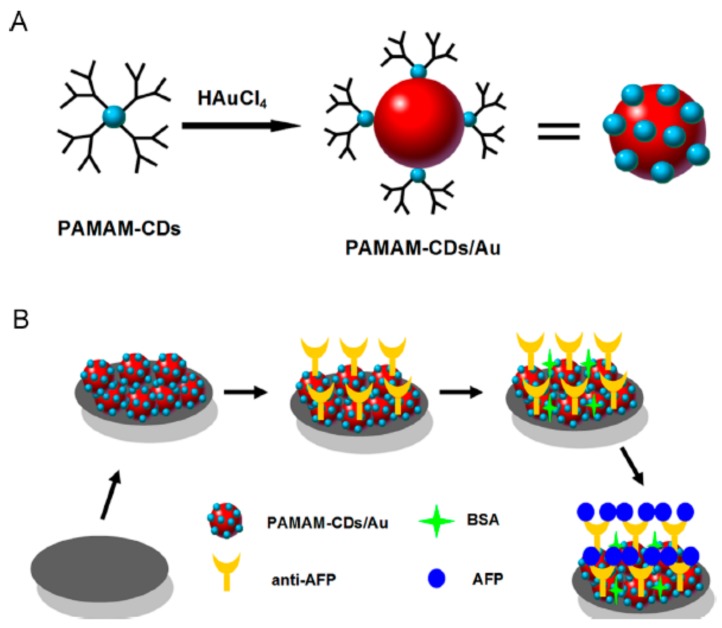
Schematic representation of the preparation of the PAMAM-CDs/Au nanocrystal hybrid nanomaterial (**A**), and fabrication of the electrochemical immunosensor for α-fetoprotein (**B**). Adapted with permission from Ref. [59], copyright Elsevier B.V., 2013.

As is schematized in Figure 9, this advanced nanohybrid was employed to construct a label-free electrochemical immunosensor for α-fetoprotein by using differential pulse voltammetry as an electroanalytical technique. This immunosensor showed a wide linear detection range from 100 fg/mL to 100 ng/mL and a detection limit of 25 fg/mL for this cancer biomarker.

Another carbon nanomaterial-based nanohybrid was reported by Bhatnagar and co-workers by combining graphene quantum dots with PAMAM G-3.0 dendrimers on Au screen-printed electrodes modified with p-aminothiophenol [60]. This electrode was employed to immobilize a specific antibody for cardiac troponin I, allowing detection of this protein with a sensitivity of 109.23 μA/cm^2^·μg and a detection limit of 20 fg/mL by using differential pulse voltammetry as an electrochemical technique.

Although far less predominant than PAMAM dendrimers, PPI dendrimers have also been employed for electrochemical immunosensors construction. As an example, Tshikalaha and Arotiba employed an electrodeposition approach to modify glassy carbon electrodes with PPI G-2.0 dendrimer and gold nanoparticles [61]. This nanostructured surface was used to construct a highly sensitive electrochemical immunosensor for the detection of cholera toxin in water samples. This device was able to detect up to 0.72 ng/L and 0.42 ng/L of toxin by using square wave voltammetry and electrochemical impedance spectroscopy as electroanalytical techniques, respectively.

**Figure 10:**
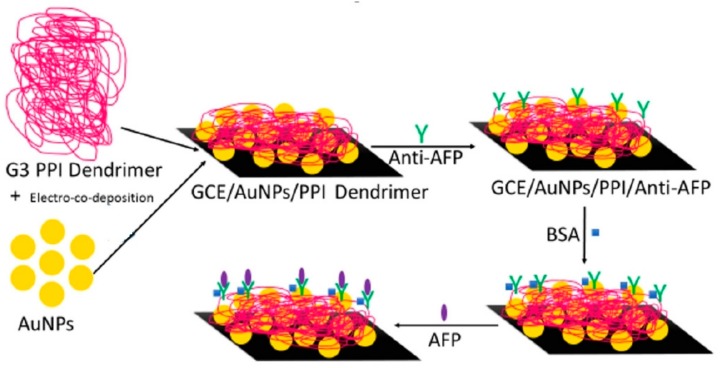
Schematic representation of the preparation of the PPI dendrimer-based electrochemical immunosensor for α-fetoprotein (AFP). Adapted with permission from Ref. [62], Copyright John Wiley & Sons, Inc., 2018.

Electrodeposition was also employed by Idris and co-workers to modify GCE with AuNP and PPI G-2.0 dendrimers to further immobilize a monoclonal antibody for α-fetoprotein [62]. This immunosensor, represented in Figure 10, was used to detect this cancer biomarker over a wide concentration range from 5 pg/mL to 500 ng/mL and detection limits of 2.2 pg/mL and 1.85 pg/mL by using square wave voltammetry and electrochemical impedance spectroscopy, respectively.

## 3. Dendrimers for Signaling and Signal Amplification in Electrochemical Immunosensors

Dendrimers bearing a redox probe at the core or at the outer surface can be employed to generate the analytical signal. In this sense, several researchers have taken advantages of the redox properties of metallodendrimers to construct label-free electrochemical immunosensors.

**Figure 11:**
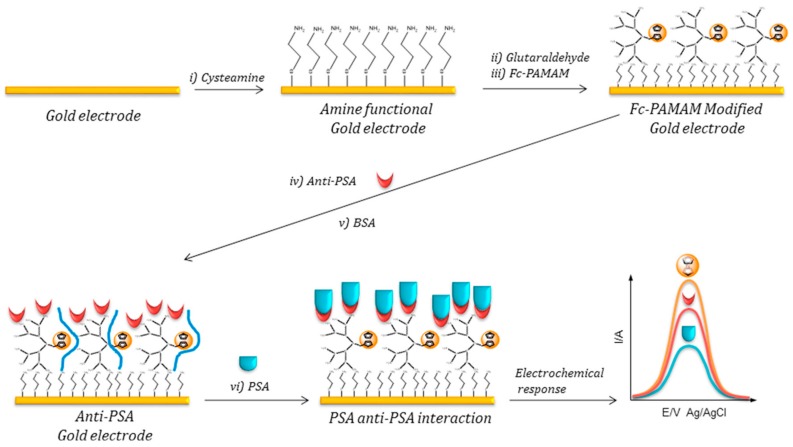
Schematic representation of the assembly steps and sensing mechanism for the electrochemical immunosensor for PSA based on ferrocene cored PAMAM dendrons. In yellow: ferrocene moieties, in red: anti-PSA antibody, in blue: PSA. Adapted with permission from Ref. [63], copyright Elsevier B.V., 2017.

Çevik and co-workers synthesized ferrocene cored PAMAM dendrons of different generations (G-1.0 to G-3.0) that were employed to coat cysteamine-modified gold electrodes through glutaraldehyde-mediated crosslinking [63]. This electrode was further employed to construct a label-free electrochemical immunosensor for the prostate-specific antigen (PSA) by covalent immobilization of an anti-PSA monoclonal antibody. As is illustrated in Figure 11, the metallodendron molecules on the electrode surface had a dual function, providing the redox analytical signal generated by the ferrocene moieties at the dendron core and the reactive primary amino groups at the surface for antibody immobilization. The sensing approach was based on the reduction of the voltamperometric signal of the ferrocene groups upon biorecognition of the analyte. The best analytical properties were achieved by the immunosensor constructed with the G-1.0 dendrons, which allowed detection of PSA from 10 pg/mL to 100 ng/mL with a sensitivity of 64.2 nA mL/ng and a detection limit of 1.0 pg/mL. A similar label-free sensing mechanism, based on redox-responsive metallodendrimers, was used to determine immunoglobulin G by employing glassy carbon electrodes coated with ferrocenyl end-grafted PAMAM G-2.0 dendrimers [64].

**Figure 12:**
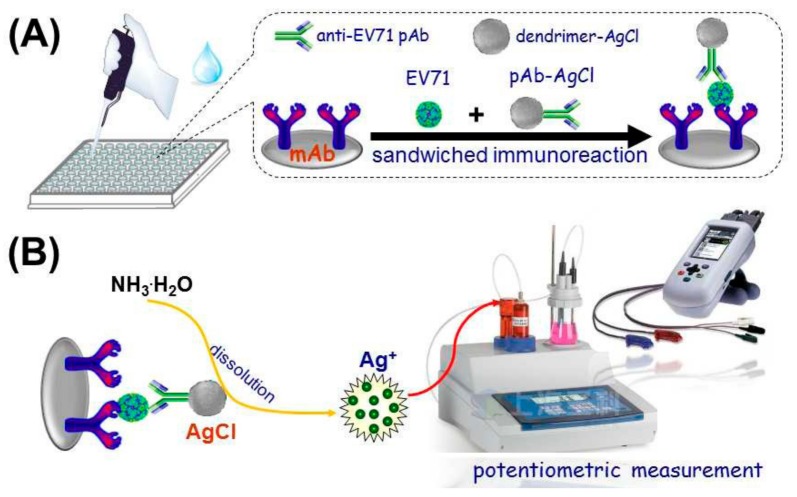
Schematic representation of the assembly steps (**A**) and sensing mechanism (**B**) for the potentiometric immunosensor for enterovirus 71. Adapted with permission from Ref. [65], copyright Elsevier B.V., 2017.

Dendrimer derivatives have also been employed for signal amplification in potentiometric immunosensors. In this field, Sun reported the preparation of trimethylol propane core, 2,2-bis(hydroxymethyl)propionic acid dendrimer G-4.0 dendrimer-doped AgCl nanospheres by a reverse micelle method [65]. This nanomaterial functionalized with a polyclonal antibody toward enterovirus 71, and the resulting adduct was employed as a labeling element for the detection of the virus through a sandwich-type immunosensing scheme (Figure 12). The electroanalytical signal was measured by using a silver ion-selective electrode after acid dissolution of the labeling AgCl nanospheres. The immunosensor developed was able to detect the enterovirus 71 in the range from 0.3 ng/mL to 300 ng/mL with a detection limit of 58 pg/mL. A similar sensing strategy was employed by Li and co-workers to construct a sandwich-type potentiometric immunosensor for α-fetoprotein, by using a CdS quantum dots-aggregated PAMAM dendrimer nanohybrid as the labeling element for the detection antibody and a cadmium ion-selective electrode as transducer [66].

**Figure 13:**
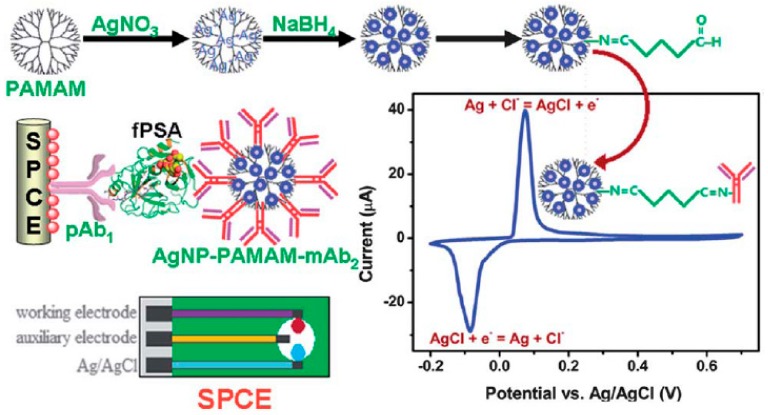
Schematic representation of the preparation of the Ag nanoparticles (blue circles)-PAMAM dendrimer nanohybrid and assembly of the immunosensor for PSA. Adapted with permission from Ref. [67], copyright The Royal Society of Chemistry, 2013.

Several nanohybrids of PAMAM and metal nanoparticles have demonstrated to be useful to design original signal amplification strategies for electrochemical immunosensors. As an example, Pei and co-workers developed a novel enzyme-free electrochemical immunoassay for PSA by using a PAMAM G-6.0 dendrimer loaded with Ag nanoparticles as a molecular tag for an anti-PSA polyclonal antibody [67]. This adduct was employed as a detection and signal amplification element in a sandwich-type immunoassay assembled on carbon screen-printed electrodes functionalized with capture anti-PSA antibodies (Figure 13). In this system, the Ag nanoparticles highly loaded into the dendrimer act as catalysts for the reduction of hydrogen peroxide in the detection solution, this producing the amplified electroanalytical signal.

**Figure 14:**
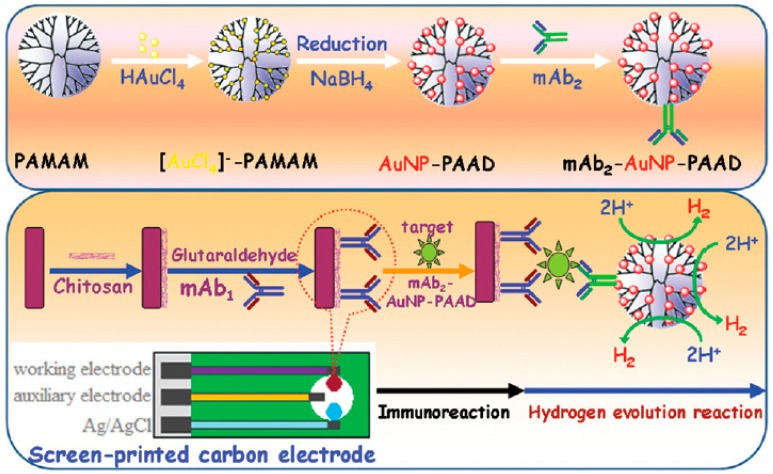
Schematic representation of the preparation of the AuNP-PAMAM dendrimer-detection antibody adduct (**top**) and assembly and performance of the immunosensor for human carbohydrate antigen 19-9 (**bottom**). Adapted with permission from Ref. [68], copyright The Royal Society of Chemistry, 2015.

By using a similar approach, Sun labeled an anti-human carbohydrate antigen 19-9 monoclonal antibody with a nanohybrid of AuNP-encapsulated PAMAM G-5.0 dendrimer to construct a sandwich-type immunosensor on a capture antibody-modified screen-printed carbon electrode [68]. As is illustrated in Figure 14, the rationale of the detection strategy was based on the electrocatalytic activity of the carried AuNP toward the hydrogen evolution reaction, allowing rapid and sensitive quantification of the target biomarker on the electrode. This immunosensor, which was validated in several human serum samples, was able to detect up to 6.3 mU/mL of the antigen. AuNP-PAMAM dendrimer nanohybrids were also employed as signal enhancement agents for the enzyme-free electroanalytical immunodetection of myoglobin by using cathodic stripping voltammetry [69].

## 4. Conclusions and Remarks

The use of dendrimers as “soft” nanomaterials in electrochemical immunosensors has been largely explored, with a great number and variety of reports already published on their use as transduction and signal amplification elements. The interest in these nanosized and hyperbranched polymers has been based on their unique structural and functional properties, as well as the possibility of using commercially available dendrimers to prepare new derivatives by chemical modification in combination with nanomaterials or functionalization with antibodies.

According to the applications outlined above, dendrimers have demonstrated to be useful as transduction elements in electrochemical immunosensors, providing high density surface reactive groups for further covalent attachment of antibodies. These hyperbranched polymers also allowed the assembly of a large variety of complex 3D architectures without a significant reduction of the electron transfer processes due to their permeable 3D structure. High solubility, controlled size and density of reactive groups, and easy modification with redox probes and other signaling elements have also allowed the design of sophisticated dendrimer-based signal amplification strategies for electrochemical immunosensors with improved analytical performance.

Future advances in dendrimer-based electrochemical immunosensors should be fueled by progress in dendrimer chemical synthesis that could provide a limitless variety of new derivatives with mixed chemical functionalities, electroactive redox probes and tuned cavities for tailored encapsulation of nanomaterials with a defined size and surface modification. Advances in nanomaterials engineering should also contribute to the tailored design of dendrimer-based nanohybrids with enhanced conductivity, redox properties and structural compatibility with antibody molecules for the construction of reliable, sensitive and stable immunosensor devices.
